# A 10-Year Journey of the USM Master and Doctoral Degrees in Public Health

**DOI:** 10.21315/mjms2019.26.6.1

**Published:** 2019-12-30

**Authors:** Surianti Sukeri, Aziah Daud, Kamarul Imran Musa, Norazlin Idris

**Affiliations:** Department of Community Medicine, School of Medical Sciences, Universiti Sains Malaysia, Kubang Kerian, Kelantan, Malaysia

**Keywords:** Master of Public Health, Doctor of Public Health, community medicine

## Abstract

The article introduces readers to the Master of Public Health and Doctor of Public Health programmes, offered by the Department of Community Medicine, School of Medical Sciences, Universiti Sains Malaysia. The programme vision, structure and accomplishments over the decade are presented to provide an understanding of the programme. It is hoped that this professional programme will continue to flourish and produce new generations of public health medicine specialist equipped with the necessary knowledge and skills to make significant contribution towards improving the health of the population.

## Special Communication

There is an oil painting adorning the wall of my office. This beautiful landscape of trees painted in hues of greens brought me the memory of the student who gifted me the painting. I remember this student well, a quaint persona, distant but not unfriendly, headstrong but very independent and despite all her personal hardship, managed to persevere and graduated from the 1+3 Master of Public Health (MPH)/Doctor of Public Health (DrPH) programme. She is one of the 48 graduates produced since the inception of the programme in 2010. Forty-eight public health physicians who have now become eminent advocators and leaders of public health medicine in Malaysia. Their achievements are our pride and joy, produced #USMstyle.

The 1+3 MPH/DrPH programme is our core business at the Department of Community Medicine; a programme designed as a response to an acute need of the Ministry of Health for Medical Officers of Health to serve in the then fast expanding rural health services. The intention of the programme is to produce competent health professionals with the appropriate knowledge and skills in public health to plan, implement and evaluate effective public health programmes that can improve the quality of life of the community. The MPH programme is a two-semester coursework degree, carrying a total of 40 units consisting of 32 core units and 8 units of Research Project (1). The DrPH, on the other hand is a public health physicians professional graduate programme. It is a 90-credit mix-mode, full time doctoral degree awarded in the field of public health (2). This programme was established to train manpower that is knowledgeable and skilful in the specialty of public health medicine and its related disciplines. It is an advanced programme meant for degree holders in medicine who aspire to develop their careers as public health physician. It focuses on the development of knowledge and skills in professional leadership, administration and the application of knowledge-centred ‘evidence-based medicine’ with the emphasis on health development and problem-solving activities at the community, national and international levels.

The DrPH programme is conducted over six semesters; students are required to pass their course work, undergo field attachment and produce a dissertation as a requirement for completion of the study. Students may choose any one of the four thrusts of public health; Epidemiology, Family Health, Occupational and Environmental Health, and Health System Management. We provide hands-on learning in analytical skills, rigorous training in research methodology and direct field experience in the community. Students are trained in evidence-based public health practice and research, are expected to have the competences to convene diverse stakeholders, communicate across a range of sectors, synthesise and disseminate findings, and generate practice-based evidence. Some see it as a tough programme, designed only for the fittest of the fit. From time to time students came to see me, tears streaming, eyes swollen. They find it hard to adapt, no longer are they treading on the familiar terrain of clinical medicine. Subjects such as Medical Sociology, Health System Management, Health Policy and Ethics are so foreign to the mind of a typical reader in medicine. However, as the world face unprecedented public health challenges such as health crises, wealth gap, climate change, non-communicable diseases, aging populations, it is crucial that our graduates should receive their fill of training that prepare them to be technically competent in the field of public health with an understanding of the relationship between social determinants of health, culture and other factors that may affect the health of the community.

**Figure 1:**
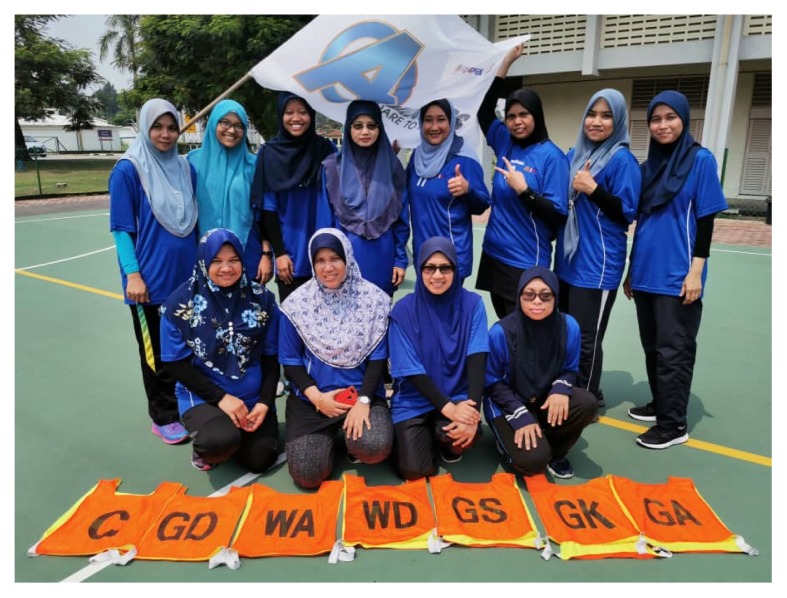
Netball tournament organised by the School of Health Sciences, USM

As future public health physicians, we groom our students to be role models to the community. We encourage our students to lead healthy lifestyle, be physically active and engage in various type of sports activities and tournaments such as futsal, netball, soccer, marathon and cycling (Figure 1).

**Figure 2:**
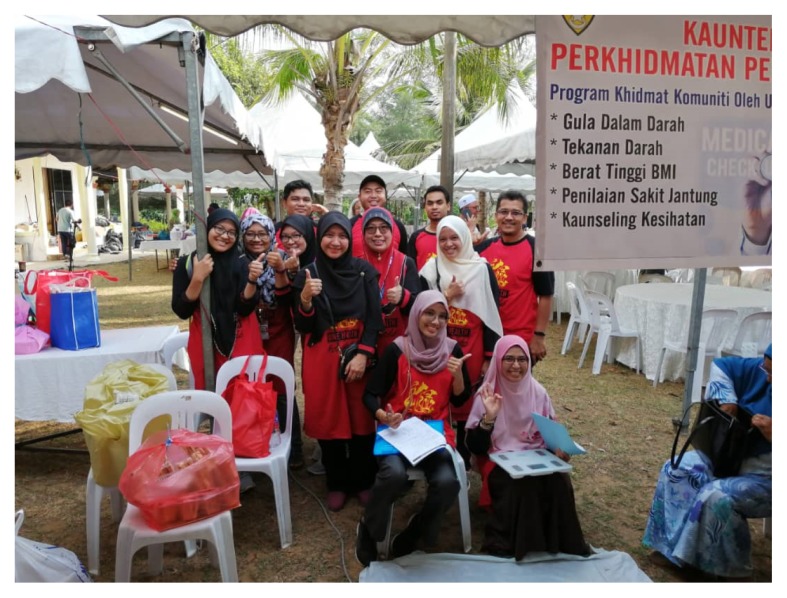
Kampung Berangan community health programme at Melawi, Bachok

Public health is a critical part of the larger concept of health systems and has been defined as ‘what we as a society do collectively to assure the conditions in which people can be healthy’ (3). Engagement with the community has always been the bread and butter for public health physicians. Our students committed in their roles, organised various community engagement programmes in rural areas to ensure the public’s access to quality health services while at the same time educating the community on the importance of maintaining healthy lifestyle (Figure 2). Health programmes are conducted regularly in schools as we believed early exposure on disease prevention is best to instil behaviours that reduce the risk of communicable and non-communicable diseases and injuries among the young.

**Figure 3:**
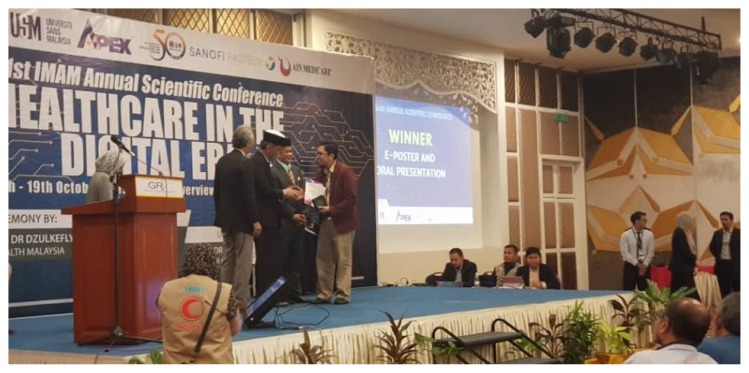
Winner of the best oral presenter at the IMAM Scientific Conference

**Figure 4:**
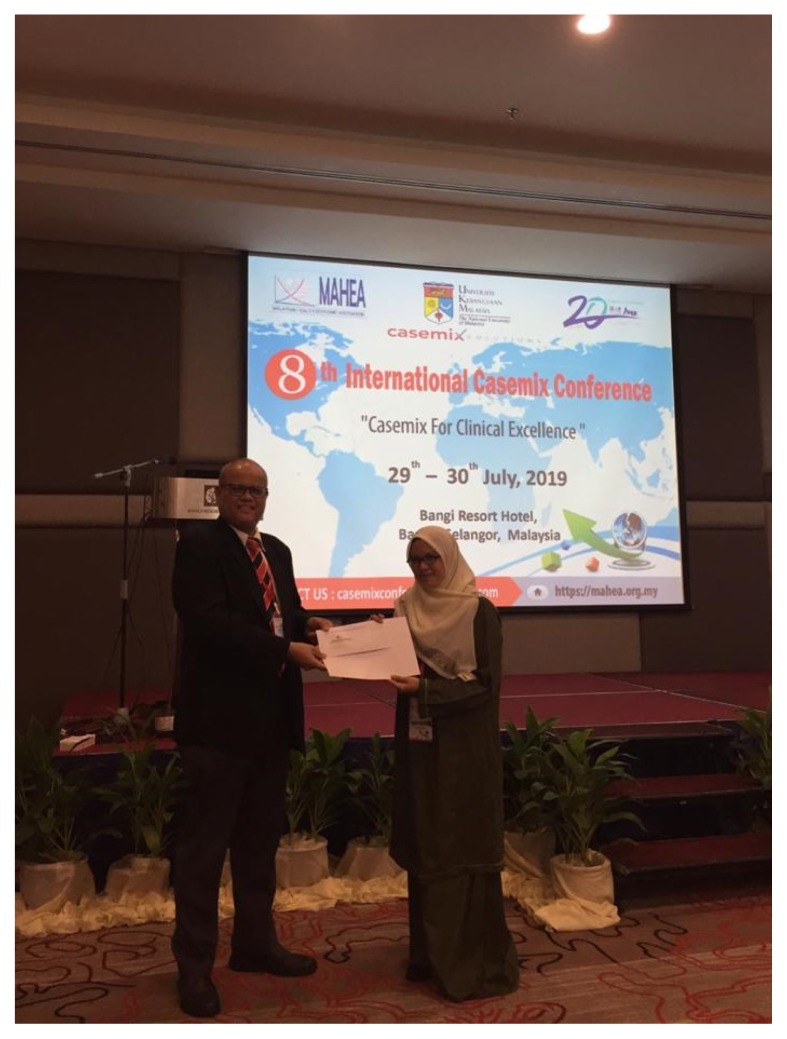
Best oral presenter at the 2nd International Health Financing Conference

After nearly one decade of the running of this programme, it is therefore proper for us to take stock and look at where we have come from, where we are today and plan for the future. We continue to encourage candidates to conduct research that fills identified gap in current public health issues and generate findings that will contribute to national and state health policy objectives. This is in line with effective translation of research outcomes primarily into practice and improvements in the health of population, patient care and public policy. The by-products of our students’ research include various scientific publications, presentations (Figures 3 and 4), books, intervention modules, questionnaire and application tools which have been accepted and adopted by researchers, policymakers and stakeholders locally, even internationally. The number of scientific publications is increasing each year, and more than 100 of these publications were published in high impact journals. We are proud to claim that many of our graduates are currently holding essential posts at district health offices, hospitals, Ministry of Health in Putrajaya and renowned institutes such as the Communicable Disease Centre in Atlanta. Lately, the programme has been garnering interests from international candidates in China, Pakistan, India and Kingdom of Saudi Arabia, a positive sign that our programme has been recognised worldwide. We pray for this excellent momentum to continue and pave the progress towards a better future of public health in Malaysia.

The success of any health system depends on the availability of an appropriately trained, competent workforce. The primary focus of public health system strengthening is to build the workforce needed to staff key national public health institutions, conduct the core functions of public health, and implement and manage critical health programmes (4). As we look upon the future, the words of Nelson Mandela indeed rings true, ‘education is the most powerful weapon which you can use to change the world’. we pray all our students, wherever they are, will continue to remember lessons we imparted to you and be the change that change the world. Remember, don’t just…

Don’t just learn, experience.
Don’t just read, absorb.
Don’t just change, transform.
Don’t just relate, advocate.
Don’t just promise, prove.
Don’t just criticise, encourage.
Don’t just think, ponder.
Don’t just take, give.
Don’t just see, feel.
Don’t just dream, do.
Don’t just hear, listen.
Don’t just talk, act.
Don’t just tell, show.
Don’t just exist, live

(Bennett, the Light in the Heart) (15)
